# FRET-Based TURN-ON Aptasensor for the Sensitive Detection of CK-MB

**DOI:** 10.3390/bios15070446

**Published:** 2025-07-11

**Authors:** Rabia Asghar, Madiha Rasheed, Xuefei Lv, Yulin Deng

**Affiliations:** 1 Beijing Key Laboratory for Separation and Analysis in Biomedicine and Pharmaceuticals, School of Medical Technology, Beijing Institute of Technology, Beijing 100081, China; 2 BIT & GS Technology Co., Ltd., Beijing 100043, China

**Keywords:** FRET, aptamer, aptasensor, MoS_2_ nanosheets, ALP, aminated graphene quantum dots (AGQDs), AGQDs@MoS_2_ nanoassembly

## Abstract

A fluorescent sandwich assay was devised to quantify CK-MB. In a typical immunoassay, antibodies bind to the target, and the detected signal is quantified according to the target’s concentration. We innovated a unique fluorescence assay known as the “enzyme-linked aptamer assay” (ELAA) by substituting antibodies with a pair of high-affinity aptamers labelled with biotin, namely apt. A1 and apt. A2. Avidin-labelled ALP binds to biotin-labelled aptamers, hydrolyzing its substrate, 2-phosphoascorbic acid trisodium salt, resulting in the formation of ascorbic acid. The catalytic hydrolysate functions as a reducing agent, causing the deterioration of MoS_2_ nanosheets. This results in the transformation of MoS_2_ nanosheets into nanoribbons, leading to the release of quenched AGQDs. The reestablishment of fluorescence is triggered by Förster Resonance Energy Transfer (FRET) between the MoS_2_ nanoribbons and AGQDs, enhancing the sensitivity of disease biomarker detection. The working range for detection falls between 2.5 nM and 160 nM, and the limit of detection (LOD) for CK-MB is verified at 0.20 nM.

## 1. Introduction

An acute myocardial infarction (AMI) is a type of ischemic heart disease characterized by a significant incidence of morbidity and mortality, rendering it one of the most immediately life-threatening medical conditions [1]. Precise recognition of AMI holds paramount importance in clinical settings. The accurate determination of biomarker concentrations serves as a highly effective method for diagnosing AMI with exceptional precision and reliability [2]. Creatine kinase MB (CK-MB) stands as a common biomarker for AMI, possessing excellent specificity and sensitivity. Maintaining continuous surveillance of its levels can be employed to anticipate the likelihood of recurrent infarction [3]. CK-MB is found in the cardiac muscles, skeletal muscles, uterus, diaphragm, small intestine, and prostate tissues. Approximately 20% [4] of the total creatine kinase (CK) is in the myoglobin (MB) form within myocardial tissue. In the event of an acute myocardial infarction, when the myocardium ruptures, CK-MB is released into the bloodstream within 30 min. The CK-MB quantity is approximately 85 kDa [5]. In the bloodstream, the CK-MB levels increase between 4 to 6 h, eventually stabilizing at 24 h, and typically fall within the range of 39–185 ng/mL [6].

Numerous techniques for the detection of CK-MB are presently under development, including SPR and SERS methods [7], radioimmunoassay (RIA) [8], lateral flow immunoassay [9], and electrochemical methods [10]. Despite its reliance on skilled personnel and automated equipment, ELISA is more often used than other procedures [11,12]. Typical ELISA kits used in clinical settings followed a basic premise of over 6 h longer incubation period adds a tedious effort [13]. However, some drawbacks of antibody-based assays (i) labor-intensive, complicated, and an expensive culture cell medium is required to create a particular antibody. (ii) A high likelihood of false-positive or negative findings due to inadequate blocking of the surface of the microtiter plate immobilized with the antigen. (iii) Antibody instability, as an antibody is a protein that must be transported and stored in a refrigerator [14], is inevitable. The present study introduces an innovative sensing approach. The substitution of antibodies with aptamers in the assay has enhanced cost-effectiveness. Aptamers exhibit stability and can be readily tailored to specific targets. These high-affinity molecules serve a pivotal role as labelled probes, directly binding to the target.

**Scheme 1:**
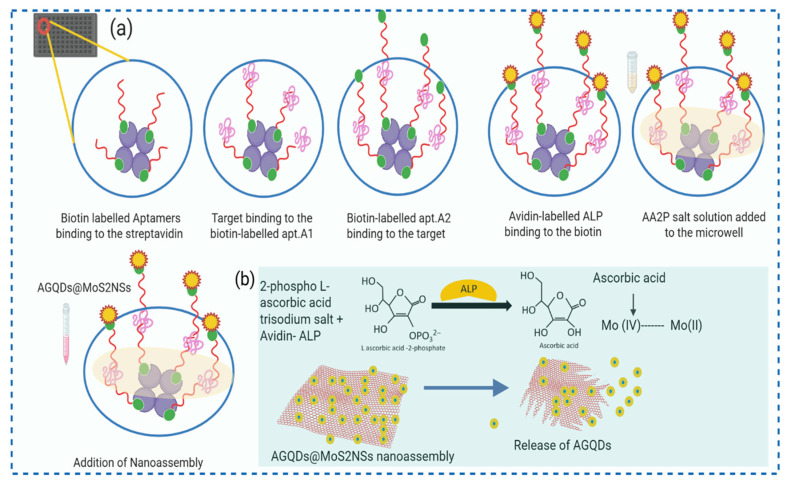
(**a**) Schematic Illustration of assay designed for the detection of CK-MB. (**b**) Representing action of enzymatic hydrolysate for the breakdown of AGQDs@MoS_2_NSs nanoassembly to release AGQDs.

Aptamer assay-based detection of CK-MB [15,16,17]. A number of studies have utilized this approach. We are introducing a unique fluorescence assay based on aptamers, which employs a nanoassembly consisting of aminated graphene quantum dots (AGQDs) and MoS_2_ nanosheets as the fluorescence probe (as presented in Scheme 1).

In this method, we leverage strong electrostatic interactions to create the nano assembly of MoS_2_ nanosheets (MoS_2_NSs) and aminated graphene Quantum dots (AGQDs). This graphene-like two-dimensional material has previously been employed as a quencher in biosensing platforms designed for the detection of nucleic acids, proteins, small molecules, and metal ions [18,19]. Because of its extremely narrow bandgap semiconductor properties and single-layer structure, it finds diverse applications in nanoelectronics, optoelectronics, and energy harvesting, among others [20]. Its single-layer structure enhances the effectiveness of electron mobility in inducing quenching. Graphene quantum dots (GQDs) exhibit superior qualities compared to organic dyes and semiconductor quantum dots (QDs), including remarkable photostability against photobleaching and blinking, biocompatibility, and low toxicity [21]. In contrast to carbon dots, graphene quantum dots (GQDs) incorporate a graphene structure, regardless of their size, which imparts to them certain distinctive characteristics of graphene [22]. The process of amination shifted the surface charge of the GQDs from negative to positive, rendering them environmentally friendly and reducing their cytotoxicity [23]. Following the creation of the nanoassembly, the Forster resonance energy transfer (FRET) interaction between AGQDs and MoS_2_ NSs will lead to the quenching of AGQD fluorescence. In our assay, we used this quenched nanoassembly for signal restoration. Concurrently, alkaline phosphatase (ALP) catalyzes the hydrolysis of 2-phosphoascorbic acid (AA2P) to generate ascorbic acid (AA). AA can induce the disintegration of MoS_2_ NSs into nanoribbons [24]. We used this enzymatic hydrolysate for FRET establishment for quenched nanoassembly. Using the signal generation approach outlined earlier, a highly sensitive aptamer-based fluorescent assay for CK-MB monitoring was effectively established. An aptamer-based fluorescent assay for very sensitive monitoring of CK-MB was successfully developed.

## 2. Materials and Methods

### 2.1. Materials

CK-MB (ProSpec-Tany TechnoGene Ltd. (Rehovot, Israel)), myoglobin, cardiac troponin I, IgG, BSA, Albumin, AFP, Hemoglobin, and Thrombin (Abcam (Cambridge, MA, USA), Biotinylated aptamers (Sangon Biotech, Shanghai, China), Bovine serum albumin (BSA), streptavidin, and Tris-EDTA buffer (Beijing Solarbio Science & Technology, Beijing, China), avidin labelled ALP (Sigma Aldrich, Shanghai, China Inc.), L-Ascorbic acid 2-phosphate trisodium salt (Adams beta, Shanghai Reagent Co., Shanghai, China), aminated graphene quantum dots N-100 (developed by bottom-up method), MnO_2_ powder (HPLC grade 99%), and MoS_2_ nanosheets (layer 1–10, developed by physical method without stabilizer) were obtained (XF nano Nanjing, China) and used without any further processing. All buffers (PBS, PBST, Coating Buffer, AP Buffer, and blocking Buffer) were prepared in distilled water.

### 2.2. Instrumentation

Multi-mode microplate readers (Cytation 3) were purchased from BioTek Instruments, Inc. (Winooski, VT, USA), and Corning® 96 Well Solid Polystyrene Microplate (Sigma Aldrich, Shanghai, China Inc.), The ultrasonic machine (KQ5200E) was purchased from Kunshan Ultrasonic Instruments Co., Ltd. (Kunshan, China Inc). Thermostatic shaking incubator (ZHWY-2102) was purchased from Shanghai Zhicheng Analytical Instrument Manufacturing Co., Ltd. (Shanghai, China). The fluorescence microscope digital camera (Olympus DP72) was purchased from Olympus Optical Co., Ltd. (Tokyo, Japan) and was used throughout the whole study. 

### 2.3. Aptamers for CK-MB (Magnetic-SELEX)

In our previous group work, aptamers for the specific target CK-MB were designed using the magnetic SELEX [25]. Further, we utilized the aptamer sequences designed in our lab on the basis of their superior binding affinity and specificity, as indicated by their low dissociation constants (Kd). Specifically, apt. A1–(5′-Biotin-GGGGGGTGGGTGGGGGATCTCGGAGGATGCTTTTAGGGGGTTGGG-3′), and apt. A2–(5′-Biotin-CATTGAGAGGGGGTGGCCGTAGTCAGGTGGGTGGGGGTTTGAG-3′ [26]. To obtain optimal binding and reduce binding competition lower Kd aptamer is used as a primary probe.

### 2.4. Preparation of Nanoassembly (AGQDs@MoS_2_NSs)

In order to observe the surface charge stability of two distinct dispersing media, distilled water and PBS buffer (pH = 7.2–7.4), the first step involved selection of the medium for the preparation of nanoassembly. The fluorescence intensity of AGQDs in both solutions was recorded by using a spectrophotometer Cytation3 (Figure S1). A clear solution of AGQDs N-100 was prepared by adding different concentrations (20 µg/mL–1000 µg/mL) to PBS buffer and distilled water. A high fluorescence intensity was recorded at an optimized concentration of 60 µg/mL in distilled water. Later on, optimal concentration of AGQDs and MoS_2_ NSs to prepare a stable nanoassembly (AGQDs@MoS_2_NSs) was obtained. MoS_2_ solution (1 mg/mL) was prepared in water: ethanol (1:1 *v*/*v*) through sonication for 30 min at 37 °C and stored at 4 °C till further use. Each concentration of AGQDs was introduced to the prepared MoS_2_ solution separately at pH 7.2 and incubated at 37 °C for 20 min. The nanoassembly is established without any centrifugation or stirring at room temperature. The fluorescence emission and UV-vis absorbance of the AGQDs@MoS_2_NSs nanoassembly were measured in the range of 400–700 nm and 280–700 nm, respectively. Different concentrations of MoS_2_ nanosheets were added to the AGQDs solution to obtain maximum quenching. The quenching efficiency (Q.E (%)) was obtained using Equation (1)

(1) $$\frac{F_{o}-F_{q}}{F_{o}}\times 100$$

where F_o_ and F_q_ are the fluorescence intensity of AGQDs and quenched fluorescence intensity after the addition of MoS_2_. For the present study, the recorded fluorescence quenching efficiency is 97% which is decreased in the presence of a higher concentration of MoS_2._

The nanoassembly with optimal values was used for further experiments.

### 2.5. Development of Sandwich Assay

A 200 µL streptavidin solution in coating buffer was added to each well of a microwells plate and incubated at 4 °C for 12 h. The wells were washed thrice with tween-20 solution in PBS (PBST) and blocked with 250 μL of 5% BSA for 1.5 h. After repeating washing 3 times, 100 μL of biotinylated-aptamer (apt. A1) solution was then added to the wells and incubated at 37 °C for 30 min. Subsequently, different concentrations of CK-MB (2.5 nM–160 nM) were added to each microwell. Following three washes, 100 μL of biotinylated aptamer (apt. A2) was added to the microwells and incubated at 37 °C for 30 min. Again, the plate was rinsed three times with PBST before adding 100 μL of avidin-ALP and incubating for 40 min. An AA2P salt solution was added, and an enzymatic reaction was allowed for 20 min. After that, a solution of already quenched nanoassembly of AGQDs@MoS_2_NSs was added, and fluorescence was observed after 30 min of incubation at room temperature.

### 2.6. Experimental Optimization of Assay Components

We optimized several assay components to assess the optimal rate of quenching. The optimized concentrations of AGQDs and MoS_2_ NSs at various time intervals (5 min to 80 min) were recorded. The initial fluorescence of AGQDs was measured before adding MoS_2_ NSs. After adding MoS_2_ NSs, plates were incubated to monitor quenching at different time intervals (Figure S2). Additionally, alkaline phosphatase was optimized against the fixed concentrations of apt. A1, apt. A2, and CK-MB (60 nM). The plates for the experiment were prepared by subsequent assay development. These optimization experiments were conducted on two different ranges of dilutions and mediums. 

The fluorescence recovery, based on the target concentration, will be determined by the avidin-labeled ALP bound to apt. A2, which acts on its substrate to generate ascorbic acid. We compared the ALP activity in these two buffers (i) AP-Buffer, a basic buffer containing 100 mM Tris-HCl, 100 mM NaCl, 5mM MgCl2, and 0.05 percent Tween-20, pH 9.5, and (ii) PBS-Buffer, a saline buffer containing pH 7.4–7.6, the range suitable for the working of ALP in a biological systems as well. The following concentrations of ALP were incubated for 30 minutes with AA2P (100 mM). The previously quenched nanoassembly was introduced into the pre-incubated solution of ALP and AA2P to observe the restoration of fluorescence. To determine the most effective concentration of the substrate AA2P, different concentrations of water-soluble AA2P (20 mM–100 mM) salt against ALP (1:2500) were evaluated. The experiment was repeated three times for precise optimization (Figure S3). Furthermore, after optimizing concentrations of AGQDs, MoS_2_, ALP dilution, and AA2P salt concentration, incubation periods of quenching and ALP activity, and selection of buffers, the aptamers were optimized by applying the entire assay against a fixed concentration of the target. We optimized the aptamers (20 nM, 40 nM, 60 nM, 80 nM, 100 nM) against the target (CK-MB 60 nM)(Figure S4).

## 3. Results

### 3.1. Optical Characteristics of Nanoassembly

**Figure 1:**
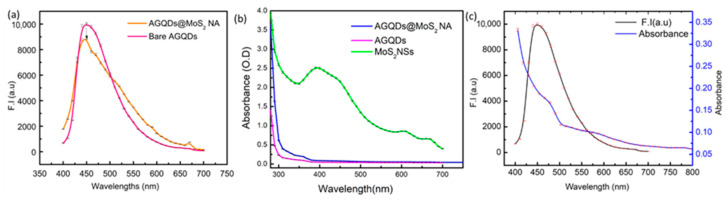
To observe successful binding of AGQDs @ MoS2 Nanoassembly. (**a**) F.I (a.u) of bare AGQDs (pink line) and AGQDs @ MoS_2_ Nanoassembly (orange line). (**b**) Absorption spectrum of bare AGQDs, MoS_2_ NSs, and AGQDs@MoS_2_ Nanoassembly. (**c**) Spectral Overlap between F.I (a.u) of AGQDs and Absorbance spectrum of MoS_2_.

The relationship between photoluminescence and optical absorbance is utilized to observe the quench pairing between AGQDs (N-100, 1 mg/mL) and MoS_2_ nanosheets (1 mg/mL). Optical absorbance of bare nanosheets was taken by using UV vis-spectrophotometer, and fluorescence spectra of AGQDs were measured. Figure 1a shows the PL intensity of AGQDs and AGQDs@MoS_2_ nanoassembly at an excitation wavelength of 379 nm. Both AGQDs@MoS_2_ and AGQDs exhibited emission peaks at a wavelength of 450 nm. The low PL intensity of the AGQDs@MoS_2_ compared to AGQDs demonstrates the successful formation of the nanoassembly and quenching ability of the MoS_2_ nanosheets.

Further, the UV-vis spectrum of bare AGQDs, MoS_2_ NSs, and AGQDs@MoS_2_ was obtained to confirm the stable nanoassembly formation. The optical characteristics of the MoS_2_ dispersion, like other typical TMDCs, consist of four trigonal prismatic characteristic peaks [27] which disappeared after the formation of nanoassembly AGQDs@MoS_2_NSs, as shown in Figure 1b. A representative of 2D nanomaterials with a large surface area and magnified ability to absorb light. MoS_2_ is particularly sensitive to light with a wavelength of 450 nm [28]. A variety of active pots that can serve the binding of carbon dots with other forms of nanomaterials to produce multifunctional nanoassemblies [29]. The decrease in fluorescence of AGQDs after the adsorption to the MoS_2_ nanosheets is a graphene dots behavior. When it covalently couples or electrostatic interactions with inert metals, metallic NPs, QDs, MOFs, LTMDs, etc. Figure 1c shows a gradual decrease in the fluorescence emission intensity at 450 nm after the formation of AGQDs@MoS_2_NSs nanoassembly, which indicates strong absorption by MoS_2_ nanosheets and confirms efficient Förster resonance energy transfer (FRET) from AGQDs to MoS_2_. There is a wide-ranging overlap of the spectra between the absorbance spectra and PL-spectra of the AGQDs observed in Figure 1c. The spectral overlap of the donor’s emission spectrum and the acceptor’s absorption spectrum is observed when photoexcited CDs (energy donor) return to the ground state orbital while simultaneously providing energy to excite other nanomaterials (energy acceptor) in the FRET system of CDs-based nanoassembly [30]. Good quantum yield is due to the photoemissive property of the fluorescent materials. The emission spectrum of AGQDs was measured between 350 and 700 nm. This spectral overlapping is an obligatory factor for the probe and quencher pairing of FRETS. The efficient quenching is due to the broad absorbance spectrum of MoS_2_ within the range of emission of AGQDs.

### 3.2. Effect of Ascorbic Acid on the MoS_2_ Nanosheets

**Figure 2:**
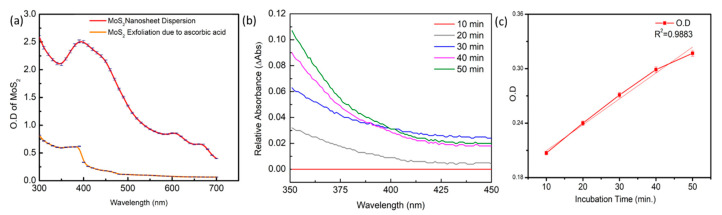
(**a**) Absorbance spectrum of the MoS_2_ NSs are shown (red curve) after ascorbic acid action, the characteristic peaks of MoS_2_ NSs (orange curve) is disappearing due to exfoliation caused by ascorbic acid. (**b**) To observe the stable enzymatic activity of ALP on AA2P to produce ascorbic acid with respect to reaction time, its absorption spectrum is obtained. (**c**) Optical density of ALP + AA2P reaction mixture measured at 350 nm at various incubation time periods is linearly increasing.

Optical characterization is applied to observe the effects of ascorbic acid on the MoS_2_ NSs. The relationship between the increased optical density of the ascorbic acid and its relative effect to cause exfoliation of MoS_2_ NSs is observed. The disappearance of characteristic peaks of MoS_2_ is visibly seen in Figure 2a. As a weak reductant, ascorbic acid exposed Mo (IV) atoms at the margins, breaking the MoS_2_NSs structure, causing unevenness, and facilitating nanoribbons production [24]. The defects in the MoS_2_ NSs desorption of the AGQDs, resulting in the release of quenched AGQDs to restore the fluorescence. The non-exfoliated MoS_2_ nanosheets showed the characteristic peaks of the MoS_2_ [Figure 2a (red curve)], which disappear after the action of ascorbic acid to deteriorate the layers of MoS_2_ [Figure 2a (orange curve)]. The UV-vis absorption spectrum of the reaction mixture containing ALP and AA2P was taken after setting the baseline at 10 min, the reaction between ALP and AA2P was observed to proceed at a constant rate up to 50 min, resulting in the generation of AA. to observe enzyme-substrate complex behavior with respect to time. The rate with respect to time goes up as the ascorbic acid is produced in the reaction mixture Figure 2b with a constant rate showing the significant production of ascorbic acid to facilitate the exfoliation of MoS_2_ nanosheets Figure 2c.

### 3.3. Optimization Experiments

The assay is for highly sensitive detection of disease biomarkers. The designed assay worked best under the determined optimal values. The performance of the current study as compared to previously reported assays is presented in Table 1. The as-developed assay had a lower LOD than most of the reported strategies. The improvement of the assay sensitivity was attributed to the successful signal amplification using the AGQDs@MoS_2_ nanoassembly as the probe.

The production of enzymatic hydrolysate ascorbic acid is relevant to the ALP-activity on its substrate. The binding event of the avidin-labelled ALP to the biotin-aptamer is according to the detected target. ALP concentration is optimized prior to determining stable activity on its substrate AA2P (Figure S4). The enzyme has a good degree of stability between pH ranges of 7.5 and 9.5, as the pH of the enzyme is affected by the substrate, its concentration, and ionic concentration [32]. While in some studies the optimal pH range for ALP activity is around 4.5, it is reported that it works significantly in a basic buffer [33]. However, considering the fact pH range and dispersion media to prepare the ALP solution might be critical for signal reading. A saline buffer outperforms an AP buffer. The enzymatic hydrolysate is an acid, Ascorbic acid, that will be neutralized in the presence of a basic medium (AP-Buffer) and will impede ascorbic acid activity to further decrease MoS_2_ nanosheets. So, a preferred medium PBS buffer (pH = 7.4) was used for the dilution of ALP (20 mU/mL).

Under the optimal conditions, ALP (1:2500) and AA2P (100 mM), the incubation period (20 min) was optimized for the ALP activity on its substrate. Further, The Mean values for apt. A1 and apt. A2 indicated that a 40 nM aptamer concentration was optimal. At higher concentrations, the linear decrease in the fluorescence restoration was found (see Supplementary Materials).

### 3.4. AGQDs and Quenching Behavior in Two Different Dispersing Mediums

**Figure 3:**
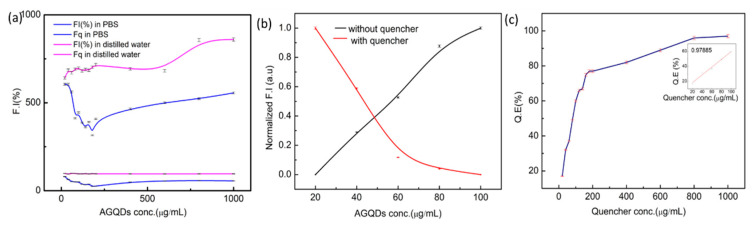
(**a**) Comparing the Fluorescence intensity [F.I (%)] and quenching efficiency of different conc. of AGQDs and quencher MoS_2_ (1 mg/mL) in distilled water and PBS Buffer. (**b**) The normalized F.I (a.u) of different concentrations of AGQDs observed with and without quencher. The black line indicating the increase in F_o_’ by the increase within the concentrations of AGQDs, while in the presence of quencher, the F_q_’ it goes on decreases. (**c**) Quenching efficiency Q.E (%) (F_o_ − F_q_/F_o_ × 100) of MoS_2_ (1 mg/mL).

Understanding the fluorescence mechanism in various solvents is critical for optoelectronic, targeted delivery of drugs, and bio-imaging applications. Under UV-light appear blue at 365nm, and near pH = 7 of the solution, the quantum dots have steady emission. However, severe acidic and basic circumstances showed a significant drop in luminescence intensity, that ascribed to the developed Lewis acids: protons (H^+^) and sodium ions (Na^+^) and Lewis bases: thiolated (S^−^) and oxygen ions (O^−^) which may cause the aggregation of quantum dots at high ionic strength [34]. As further application of alkaline-based enzyme (ALP) in the assay may interfere under the acidic or alkaline pH of buffers. Due to the presence of functional groups, GQDs are well diffused in several solvents, including water [35,36]. Edge fractures and functional groups of nano-sized GQDs are governed towards the exterior because GQDs are ultra-small graphene fragments. Because of their plethora of defect sites and functional groups, GQDs offer a particular advantage over other nanostructured materials [37,38,39,40]. Utilizing these concepts aminated graphene quantum dot behavior in two different mediums was observed. In Figure 3a, the fluorescence intensity of different concentrations of AGQDs was quenched by MoS_2_ nanosheet dispersion (1 mg/mL). AGQDs at an optimized concentration of 60 µg/mL in water-based medium showed the optimal quenching efficiency. The results showed that aqueous solutions have brighter illumination than the solution prepared in PBS buffer (pH = 7.4). The diameters of GQDs are affected to some extent by the solvents employed in GQD synthesis [41]. A nonlinear trend found at high AGQD concentrations, self-absorption, or the inner filter effect may reduce fluorescence, although increased fluorophore density can eventually dominate. Aggregation at higher concentrations can alter quantum yield or scattering properties. Additionally, the ionic strength or pH of the buffer may affect AGQD colloidal stability by shielding surface charges.

MoS_2_ is an excellent quencher against several fluorophores. 2D material nanosheets attract the biomolecules, fluorophores, due to van der Waal’s force or other non-covalent forces. In Figure 3b, the normalized fluorescence intensity of AGQDs, measured both with and without the quencher, showed a consistent pattern between the donor and acceptor. The quenching efficiency ranges between 95% and 97.7% in the previous investigations [42]. The quenching attains equilibrium after reaching a certain concentration limit. In Figure 3c, the maximum quenching shown was selected as the critical parameter. As the concentration of quencher against the optimized concentration of AGQDs increases, the Q.E% increases.

### 3.5. Effect of Substrate on Enzyme Activity

**Figure 4:**
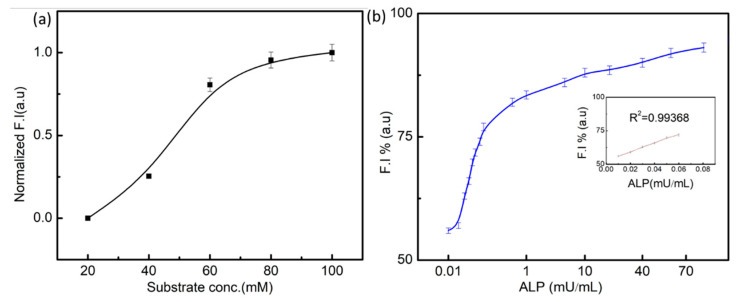
(**a**) Effect of substrate concentration on the enzymatic reaction between ALP and AA2P (20 mM, 40 mM, 60 mM, 80 mM, and 100 mM). (**b**) Enzymatic activity under different concentrations of enzyme against a fixed concentration of substrate.

The hydrolysis of AA2P by the enzyme exhibited a hyperbolic relationship with increasing concentration of substrate, and no inhibition by excess of substrate was observed, as shown in Figure 4a. The specific activity of the enzyme for the hydrolysis of its typical substrate was 113.5 U/mg and K0.5 = 65 μM. Kinetic data revealed that the hydrolysis of PNPP exhibited cooperativity with n = 1.3 [43]. The concentration and ionic concentration of substrate affect the activity of ALP. By using optimal concentrations of substrate, enzyme action is measured at different concentrations of ALP (0.01–100 mU/mL). The probability graph of the ALP concentration is presented in Figure 4b. There was a linear relationship between fluorescence intensity (restored) and ALP concentrations (F = 52.+000 [ALP] (mU/mL)R^2^ + 0.9936). The present assay has higher sensitive results for the detection of ALP as compared to the fundamental colorimetric assay.

### 3.6. Stability of Sensor

**Figure 5:**
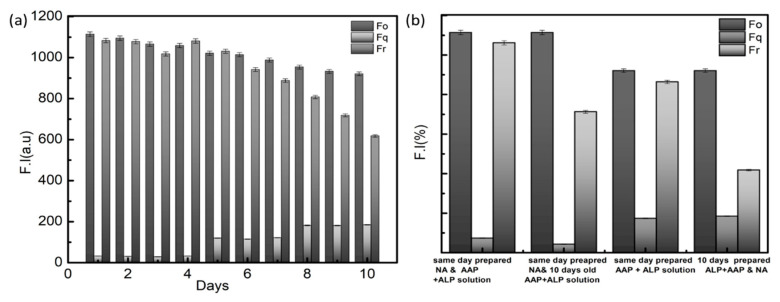
(**a**) Fluorescence restoration [F.I (a.u)] stability within consecutive ten days. (**b**) Stability of ALP –AA2P complex and AGQDs@MoS_2_ Nanoassembly to restore the fluorescence intensity [F.I (a.u)] against freshly prepared or ten-day-old prepared solutions.

To assess sensor reliability, produced nanoassemblies were evaluated by continually measuring fluorescence restoration over 10 days Figure 5a. Even on the tenth day, the quenching and restoration of fluorescence retains more than 50% of the original fluorescence. That is, adsorption of AGQDs on MoS_2_ nanosheets is a stable function, as is detachment of AGQDs from nanosheets. Further, A freshly prepared ALP/AA2P combination was evaluated against freshly prepared nanoassembly and ten-day-old nanoassembly Figure 5b. Similarly, a ten-day-old ALP-AA2P combination was incubated against a newly generated nanoassembly and a ten-day-old nanoassembly. All of the mixes performed far over 50%. Even the tenth old mixes. The stable action of ALP-AA2P to produce ascorbic acid and ascorbic acid to corrode MoS_2_ nanosheets has a stable performance.

### 3.7. Analytical Performance of the Assay

In determining the sensitivity of the sandwich fluorescence test. The standard curves for the detection of CK-MB were created under optimal settings.

**Figure 6:**
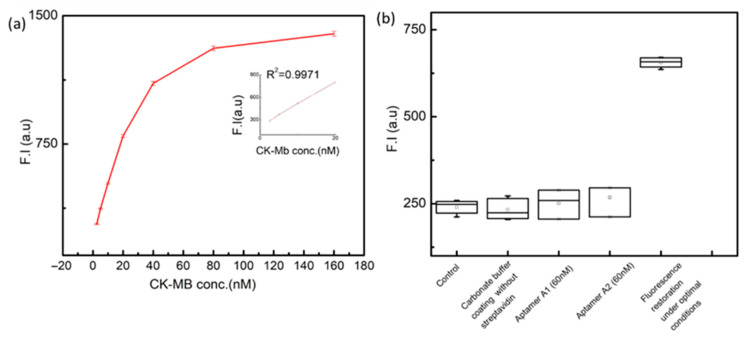
(**a**) The quantitative detection and qualitative analysis of CK-MB by fluorescent aptamer-based assay with a series of spiked concentrations (0 nM, 2.5 nM, 5 nM, 10 nM, 20 nM, 40 nM, 60 nM, 80 nM, 160 nM) in artificial serum. Calibrated curve for the quantitative detection of R^2^ = 0.9971 is presentinged. All values are means ± SD (n = 3). (**b**) Different parameters of the as-say were controlled for the specificity of the assay developed ion the plate.

To calculate the LOD of the assay experiment was repeated three times, and also run the control experiments to take the mean of the blank experiments. The LOD was to be calculated by using (LOD = 3σ/S). The linear regression curve for the detection of CK-MB was developed under optimized conditions and is shown in Figure 6a. A high-sensitivity fluorescence signal output is successfully read out using AGQDs@MoS_2_ as the probe. The innovative fluorescent assay has a LOD of 0.20nM. The simplest regression model is applied to observe the relationship between X (CK-MB known concentrations) and Y (signal output) is a straight line, Y = ax + b, where a is the y-intercept and b is the slope of the line. The regression equation can be fit into a polynomial as y = 29.127x + 220.43 with R^2^ = 0.9971, where x is the concentration of CK-MB, a is the angular coefficient representing the sensitivity, while b corresponds to the background signal. The fluorescence assay’s analytical performance was greatly enhanced.

In the presented study, we uniquely specify it by comparing the concentrations of the coating plate with and without streptavidin, aptamer apt. A1 (60 nM) in the absence of apt. A2 and in the absence of apt. A1 and apt. A2 (60 nM), and running the assay under the optimal conditions. The distributional parameters were compared with empty wells of fluorescent plates. A box plot is applied here to show overall patterns of response for different parameters, which are critical for a developed assay. This gives a visual range and other characteristics of responses against the only set parameter of fluorescence restoration. The results against the first four parameters suggest that overall signal strength is reduced. While for the fluorescence restoration under optimal conditions, the signal strength is maximum with a short mean difference. The devised assay employing the aptamer displays high specificity towards the target protein, with limited cross-reactivity and interference from non-target molecules, making it a reliable tool for precise detection of CK-MB and a longer shelf-life.

## 4. Discussion

The technique of antibody-antigen conjugation has been used extensively in research. Aptamers were used to detect cTnI [44,45], cTnT [46], and CK-MB [16]. Numerous investigations have been conducted to detect CK-MB. In this current study, we employ a unique sensing approach that utilizes various methods. Our developed fluorescent test exhibited a lower limit of detection (LOD) than the majority of the previously reported techniques.

The effective signal amplification, achieved by using AGQDs@MoS_2_NSs nanoassembly as the probe, can be attributed to enhanced assay sensitivity. This is due to the high molar extinction coefficient and broad absorption spectra that successfully quench AGQDs’ fluorescence through FRET. Additionally, the high oxidative capability of MoS_2_ NSs, along with ALP’s hydrolysis of AA, could reduce MoS_2_ NSs and restore AGQDs’ fluorescence. Consequently, we successfully created a fluorescent test with a high signal-to-noise (S/N) ratio, significantly enhancing sensitivity.

Furthermore, replacing antibodies with aptamers is an innovative aspect of this assay. To the best of our knowledge, such assays for CK-MB detection have not been previously established. This test can be applied to monitor CK-MB levels in serum. The incorporation of aptamers in the test, instead of antibodies, enhances cost-effectiveness. Aptamers are both stable and easily customizable for the specific target. High-affinity molecules play a crucial role as tagged probes, directly binding to the target.

By incorporating two aptamers, one for the target and one for the linkers, we devised a sandwich scheme for the assay. On the other hand, the Avidin-labelled ALP conjugated to apt. A. 2 provides a direct method to quantify the target based on its activity with respect to its substrate. The stability of the sensor has been observed, which is a crucial factor for developing assays aimed at commercial applications. The enzyme-linked activity in this assay, relying on aptamer conjugation, can be extended to the detection of various biomarkers.

## 5. Conclusions

To address the shortcomings, the emerging fluorescence immunoassay proposed the latest nanoassemblies and application of aptamers in detection coupled with simultaneous enhancement of sensitivity, shelf life and flexibility against various analytes present in the current technologies. Those traits renders the effectiveness of the assays in research besides that of clinical diagnostics.

## Figures and Tables

**Table 1:** Comparative analysis of previously reported assays for the aptamer-based detection of CK-MB.

Methods	Probes	Material	Linear Ranges/LOD	Ref.
Aptamer-based fluorometric lateral flow assay	Aptamers C.Apt.21 and C.Apt.30	Fluorescently labelled nanoparticles	0.63 ng/mL	[26]
Aptamer-based microfluidic electrochemical biosensor	Aptamers	Microfluidic chip	2.4 pg/mL	[10]
Aptamer-based lateral flow assay	Aptamers	Colloidal Gold Chromatography Strips	82 nM, 7146 ng/mL	[31]
Aptamer-based sandwich assay	Aptamers	AGQDs@MoS_2_ nanoassembly	0.20 nM, 17 ng/mL	This study

## Data Availability

All available data are mentioned within the manuscript.

## Supplementary Materials

The following are available online at https://www.mdpi.com/article/10.3390/bios15070446/s1. Figure S1: Fluorescence Intensity of AGQDs at different conc. in a distilled Water and PBS buffer. Figure S2: Incubation of the time period for the efficient quenching of fluoresce of AGQDs (60 μg/mL). Figure S3: Activity of ALP on AA2P. The relevant fluorescence restoration with respect to the produced ascorbic acid is shown [A] The fluorescence intensity changing of the sensing system to different dilutions of ALP in PBS buffer (1:70,000, 1:10,000, 1:80,000, 1:6000, 1:4000, 1:2000). The plot of the fluorescence intensity versus ALP dilutions. (λexc = 492 nm and λem = 520 nm). [B] The fluorescence intensity changing of the sensing system to different dilutions of ALP in PBS buffer (1:5000, 1:2500, 1:2000, 1:1250, 1:1000). The plot of the fluorescence intensity versus ALP dilutions. (λexc = 492 nm and λem = 520 nm). [C] The fluorescence intensity changing of the sensing system to different dilutions of ALP in PBS buffer (1:70,000, 1:10,000, 1:80,000, 1:6000, 1:4000, 1:2000). The plot of the fluorescence intensity versus ALP dilutions. (λexc = 492 nm and λem = 520 nm). [D] The fluorescence intensity changing of the sensing system to different dilutions of ALP in AP buffer (1:5000, 1:2500, 1:2000, 1:1250, 1:1000). The plot of the fluorescence intensity versus ALP dilutions. (λexc = 492 nm and λem = 520 nm). Figure S4: Optimization of incubation time required for the active and stable action of ALP on its substrate AA2P. Figure S5: Optimization of Aptamers.

## Abbreviations

CK-MB	Creatine Kinase Myocardial Band
ELAA	Enzyme Linked Aptamer Based assay
apt. A1	Aptamer A1
apt. A2	Aptamer A2
FRET	Förster Resonance Energy Transfer
Fluorescence Intensity	(F.I (a.u))
MoS_2_NSs	Molybdenum disulfide nanosheets
AGQDs	Aminated Graphene Quantum Dots
AA2P	L-Ascorbic acid 2-phosphate
AA	Ascorbic acid
AGQDs@MoS_2_NSs	Nanoassembly of Aminated Graphene Quantum dots & Molybdenum disulfide nanosheets
CD	Carbon Dots
QD	Quantum Dots
GQD	Graphene Quantum Dots
