# Supplementary material for: FRET-Based TURN-ON Aptasensor for the Sensitive Detection of CK-MB
Source: Biosensors (Basel). 2025 Jul 11;15(7):446. doi: 10.3390/bios15070446 (PMC12293767; doi:10.3390/bios15070446)
Supplement: Supplementary file 1 [file biosensors-15-00446-s001.zip › biosensors-3686741-supplementary.pdf]

biosensors

MDPI

Supplementary

FRET-Based TURN-ON Aptasensor for the Sensitive Detection of CK-MB

Rabia Asghar [1], Madiha Rasheed [1], Xuefei Lv [1] and Yulin Deng [1,2,*]

[1] Beijing Key Laboratory for Separation and Analysis in Biomedicine and Pharmaceuticals, School of Medical Technology, Beijing Institute of Technology, Beijing 100081, China; rabiaasghar@bit.edu.cn (R.A.); madiharasheed@bit.edu.cn (M.R.); xuefeilv@163.com (X.L.)

[2] BIT&GS Technology Co., Ltd., Beijing 100043, China

* Correspondence: deng@bit.edu.cn

Optimization Experiments

Optimization of AGQDs: As a whole the assay is involved the cascade of biomolecules, chemicals, and reagents. Optimization of each step is done to precise the analytical results of the assay. Starting from the nanoassembly and quenchers. The optimization of preparation medium of nanoassembly was performed in two different dispersing medium distilled water and PBS buffer (pH=7.2-7.4) by dissolving different concentrations of AGQDs (20μg/mL, 30μg/mL up to 1000μg/mL) into water and PBS (pH=7.4). The fluorescence intensity of both solutions were recorded by using spectrophotometer Cytation3 after incubating at 37°C for 20 min. **(Figure S1).**

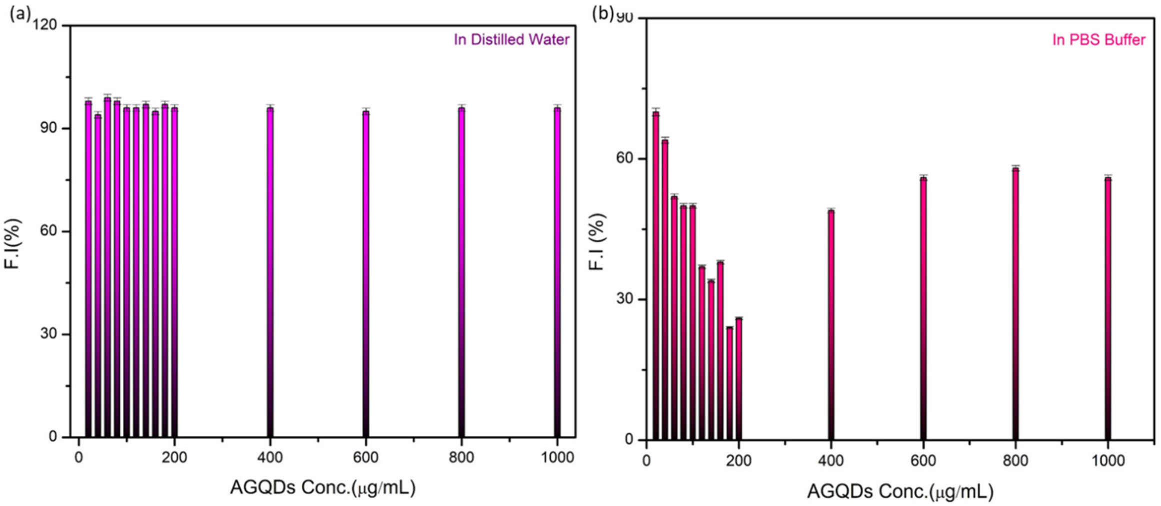

Figure S1. Fluorescence Intensity of AGQDs at different conc. in a distilled Water (a) and PBS buffer (b).

Optimization of Quenching efficiency*:* As stated earlier in main manuscript. Different concentrations of MoS_2 nanosheets were added to the AGQDs solution to obtained maximum quenching. The quenching efficiency (Q.E (%)) was obtained using equation 1 $\frac{F_o - F_q}{F_o} \times 100(1)$ where F_o and Fq are fluorescence intensity of AGQDs and quenched fluorescence intensity after the addition of MoS_2. For the present study the recorded

fluorescence quenching efficiency is 97 % which is decreased in the presence of higher concentration of MoS_2.

Optimization of incubation time of quenching: To find the optimal quenching results incubation time period were determined by incubating the optimized concentration of AGQDs and MoS_2 at different incubation period of time (5min to 100min). The initial fluorescence of AGQDs were taken before addition of MoS_2 NSs.

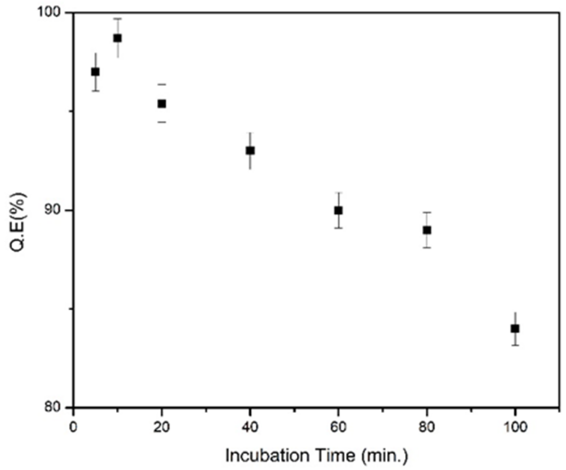

Figure S2. Incubation of the time period for the efficient quenching of fluoresce of AGQDs (60µg/ml).

Optimization of ALP concentration: To evaluate the best rate of quenching at different times. The enzymatic action between ALP and its substrate salt AAP results the formation of ascorbic acid. The fluorescence recovery according to target concentration will be determined as per the avidin labeled ALP actively work on its substrate to produce ascorbic acid. The optimized concentrations of ALP were incubated for 20min with AA2P (100mM). Later on incubated solution of ALP+AA2P were added to the already quenched nanoassembly.

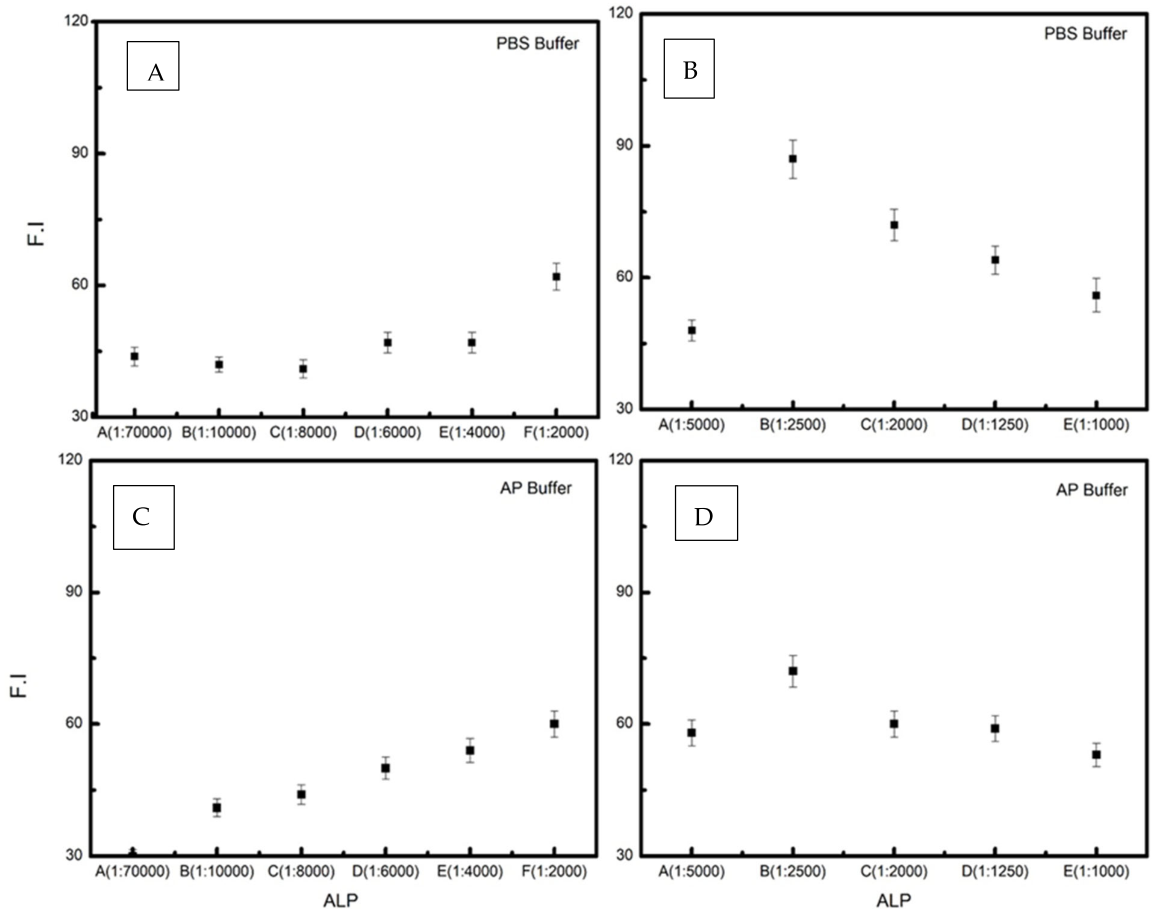

Figure S3. Activity of ALP on AA2P. The relevant fluorescence restoration with respect to the produced ascorbic acid is shown.**[A] The fluorescence intensity changing of the sensing system to different dilutions of ALP in PBS buffer (1:70000, 1:10000, 1:80000, 1:6000, 1:4000, 1:2000).** The plot of the fluorescence intensity versus ALP dilutions. (λexc = 492 nm and λem = 520 nm).**[B] The fluorescence intensity changing of the sensing system to different dilutions of ALP in PBS buffer (1:5000, 1:2500, 1:2000, 1:1250, 1:1000).** The plot of the fluorescence intensity versus ALP dilutions. (λexc = 492 nm and λem = 520 nm).**[C] The fluorescence intensity changing of the sensing system to different dilutions of ALP in PBS buffer (1:70000, 1:10000, 1:80000, 1:6000, 1:4000, 1:2000).** The plot of the fluorescence intensity versus ALP dilutions. (λexc = 492 nm and λem = 520 nm).**[D] The fluorescence intensity changing of the sensing system to different dilutions of ALP in AP buffer (1:5000, 1:2500, 1:2000, 1:1250, 1:1000).** The plot of the fluorescence intensity versus ALP dilutions. (λexc = 492 nm and λem = 520 nm).

Incubation Period and Aptamers: Under the optimal conditions, ALP (1:2500) and AAP (100mM) the incubation period (20min.) was optimized for the ALP activity on its substrate. The incubation period is equally crucial since enzymatic activity at a given time would be at best but less and more time duration may inhibit the activity (**FigureS4**).

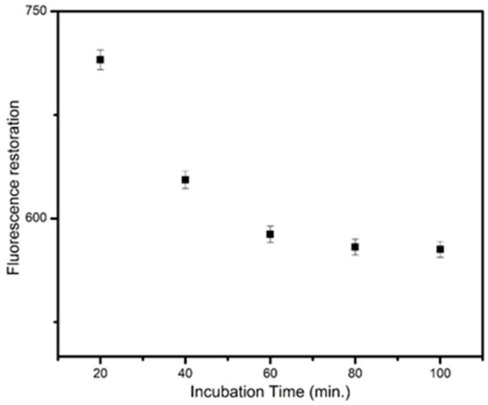

Figure S4. Optimization of incubation time required for the active and stable action of ALP on its substrate AA2P.

Optimization of Aptamers: The aptamers were optimized by applying whole assay against the fixed concentration of target. We optimized the aptamers (20nM, 40nM, 60nM, 80nM, 100nM) against target (CK-MB 60nM) and fluorescence restoration were recorded by using spectrophotometer.

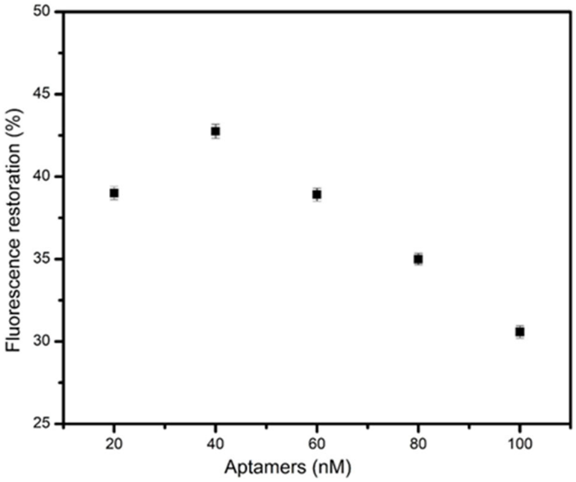

Figure S5. Optimization of Aptamers.
